# Improved beta-amyloid PET reproducibility using two-phase acquisition and grey matter delineation

**DOI:** 10.1007/s00259-018-4140-y

**Published:** 2018-08-29

**Authors:** Otakar Belohlavek, Monika Jaruskova, Magdalena Skopalova, Gabriela Szarazova, Katerina Simonova

**Affiliations:** Department of Nuclear Medicine – PET Centre, Na Homolce Hospital, Roentgenova 37/2, 150 30 Prague 5, Czech Republic

**Keywords:** Positron emission tomography, Amyloid, Brain, Dementia, Reproducibility, Flutemetamol

## Abstract

**Purpose:**

We investigated whether the reproducibility of standard visual reporting (STD method) in flutemetamol (FMM) PET can be improved using a newly introduced method that uses grey matter edges derived from the perfusion phase (GM-EDGE method).

**Methods:**

Two-phase FMM PET was performed in 121 patients with mild cognitive impairment. Five nuclear medicine physicians blindly and independently evaluated all late-phase scans, initially employing the STD method and later the GM-EDGE method. A five-point scale was used to express the degree of amyloid positivity, and a binary classification (positive/negative) was used in combination with subjective confidence (five-point scale). Multirater Fleiss’ kappa, intraclass correlation coefficient (ICC) and inter-rater reliability (Cohen’s kappa) were determined for the STD and GM-EDGE methods.

**Results:**

The weighted Cohen’s kappa values for the five-point measure of amyloid positivity ranged from 0.63 to 0.73 (median 0.70) for the STD method and from 0.76 to 0.89 (median 0.80) for the GM-EDGE method (ICC 0.84, 95% CI 0.79–0.88, for the STD method; 0.91, 95% CI 0.89–0.94, for the GM-EDGE method). The nonweighted Cohen’s kappa value for the binary classification ranged from 0.73 to 0.93 (median 0.82) for the STD method and 0.90 to 0.97 (median 0.93) for the GM-EDGE method (Fleiss’ kappa 0.82, 95% CI 0.77–0.88, for the STD method; 0.93, 95% CI 0.87–0.99, for the GM-EDGE method). The GM-EDGE method resulted in significantly greater subjective confidence in the readings of four physicians (*p* < 0.010). The binary classification was concordant among all five physicians in 80.8% of the scans using the STD method and in 91.6% of the scans using the GM-EDGE method (*p* = 0.016).

**Conclusion:**

The newly introduced GM-EDGE method was associated with significantly higher inter-rater agreement among physicians and higher subjective confidence in the reading. The method is easy to implement in clinical practice, especially when the perfusion phase is utilized clinically.

## Introduction

^18^F-Labelled beta-amyloid PET tracers, including florbetaben, florbetapir and flutemetamol (FMM), have successfully crossed the gap between research laboratories and routine clinical practice. Nevertheless, specific training of physicians is required according to the Summary of Product Characteristics (SPC) for tracers authorized for marketing in European countries (Neuraceq, Amyvid, Vizamyl). The aim of training is to guarantee reliable interpretation of PET brain scans in view of the difficulty in distinguishing specific pathological uptake in the grey matter from nonspecific physiological uptake in the white matter. This is especially difficult in the presence of brain atrophy, when the grey matter ribbon is thin and the sulci are flat.

Various approaches have been proposed to facilitate reporting and/or to quantify beta-amyloid load in the brain. They include registration of PET and MRI data, in which regions of interest in the grey matter are defined manually or using various automated approaches [1–3]. Other PET-only approaches use spatial normalization to a brain template [4] including adaptive template registration [5, 6]. The value of partial volume correction has also been considered [7] as well as standardizing quantitative amyloid PET using a centiloid scale [8]. The field is open for the application of artificial intelligence as well [9]. All these approaches are complex and they are predominantly intended for use in research. Routine clinical practice still relies on subjective visual scoring defined in the SPC.

Some authors have reported the use of beta-amyloid PET tracers for the assessment of brain perfusion [10–13]. For this purpose, supplementary data are acquired several minutes after administration of the radiotracer (early phase), when the signal from the brain represents mainly the perfusion of grey matter. In addition to the clinical benefit of the combined evaluation of brain perfusion and amyloid positivity, it is possible to easily extract the borders of the grey matter by thresholding the perfusion images, and then to superimpose these borders presented on the isocontour colour scale onto late-phase images. This facilitates delineation of white/grey matter borders and thus the determination of late-phase FMM uptake in the grey matter, which is related to the beta-amyloid load. We introduced this method [14] as an easy-to-use method, easily applicable in routine clinical practice.

The aim of this work was to assess inter-rater agreement with the standard FMM PET evaluation according to the SPC (STD method) [15] for Vizamyl as well as with the new method of evaluation involving superimposition of the grey matter borders onto late-phase images (GM-EDGE method).

## Materials and methods

### Patients

Over a period of 20 months (August 2016 to April 2018), 121 FMM PET/CT investigations were performed in 121 consecutive patients for clinical indications fulfilling the following criteria for reimbursement from the national health insurance system: for evaluation of possible Alzheimer disease (AD) not proven by National Institute on Aging-Alzheimer’s Association criteria [16], stable or progressive unexplained mild cognitive impairment or progressive dementia with atypical initial phase, for differential diagnosis (especially frontotemporal dementia), and for definition of dementia type in ambiguous cases when interpretation of clinical evaluation was not possible. Patients with clinically proven AD, patients undergoing assessment of dementia burden, asymptomatic subjects with a positive family history or ApoE genotype, and patients subjectively suffering from memory impairment without positive neuropsychological investigation were not included.

### PET/CT acquisition

A gross activity of 206.7 ± 12.7 MBq of FMM (Vizamyl; GE Healthcare) was measured 3.9 ± 3.0 min before dose administration. A noncontrast low-dose CT scan was performed for attenuation correction in the PET/CT scanner (Biograph 40 TrueV HD; Siemens). A PET list-mode acquisition was performed starting at the time of FMM administration. Data were acquired for 8 min and rebinned to dynamic datasets of 4 × 2 min without attenuation correction for motion checking only. The early-phase images were initially iteratively reconstructed to a 168 × 168 matrix, with three iterations, 21 subsets, zoom 2 and Gaussian filter 2 mm using attenuation, scatter and point spread function correction. After an uptake time of 90.6 ± 5.1 min, late-phase data were acquired for 20 min and reconstructed to a 168 × 168 matrix, with the other parameters as given above including rebinning into dynamic sequences for motion checking. After initial experience with the first 25 investigations, we shortened the late-phase acquisition time from 20 min to 10 min due to frequent patient motion in the late phase of the acquisition. We also changed the reconstruction matrix from 168 × 168 to 128 × 128 (with the other parameters as given above) to achieve a low level of noise without apparent loss of diagnostic image quality.

### Observers

Five physicians (referred to as A, B, C, D and E) certified in nuclear medicine with 5–14 years of experience in PET/CT retrospectively blindly and independently evaluated all 121 FMM PET scans extracted from the PACS in two independent runs. However, their previous experience with amyloid FMM PET reporting varied: 121, 93, 10, 0 and 0 cases, respectively. All investigators had successfully completed training prescribed in the SPC [15] 1 year previously.

### Image analysis

The Siemens *syngo*.via MM Reading tool was used for image analysis. Patient studies were arranged alphabetically. In the first run, only the late-phase images were evaluated using the STD method reported in the SPC [15] and the colour scale “Spectrum”, i.e. images were visually interpreted by comparing the activity in the cortical grey matter with the activity in the adjacent white matter. A region was considered abnormal if the tracer signal in cortical regions appeared high, i.e. approximately the same or higher than the signal intensity in the adjacent white matter and higher than that in the grey matter-rich regions of the cerebellum. If any one of following regions was clearly abnormal, then the finding was classified as positive for beta-amyloid: frontal lobes and lateral temporal lobes, anterior and posterior cingulate, precuneus, striatum and temporoparietal areas and the insula.

The instructions to start the second read were released 6 days after the start of the first read. In the second run, both early-phase and late-phase reconstructed volumes were mutually registered and oriented using the Siemens *syngo*.via MM Reading tool. Early-phase images were presented in the “Edges” isocontour colour scale and the threshold was set to delineate a thin superficial brain ribbon that mainly represented grey matter (GM). Late-phase images were presented in the “Spectrum” colour scale and the upper threshold was adjusted to 90% in the pons and cerebellar peduncles. Alpha blending of early-phase edge images with late-phase images was done by manually adjusted the mixing ratio (GM-EDGE method). Beta-amyloid-specific uptake in the delineated grey matter was evaluated in the same way as in the first run, but without knowledge of the previous result. Typical examples of image processing are discussed below.

Each physician reported beta-amyloid positivity using the STD and GM-EDGE methods in two ways. First, a five-point ordinal numerical scale was used: *1* clearly negative, *2* probably negative, *3* ambiguous, *4* probably positive, and *5* clearly positive. Second, the scans were classified in a binary fashion as negative or positive, together with the application of a five-point ordinal numerical scale expressing the subjective level of confidence: *1* very low, *2* low, *3* intermediate, *4* high, and *5* very high.

### Statistical analysis

The intraclass correlation coefficient (ICC) as a measure of absolute agreement of the ratings for one typical single rater (single measures) was determined for the five-point scale of beta-amyloid positivity using the STD and GM-EDGE methods. Additionally, inter-rater agreement (linearly weighted Cohen’s kappa) was calculated for the STD and GM-EDGE methods in physician pairs. Fleiss’ fixed-marginal multirater kappa was calculated for the binary classification of beta-amyloid positivity. In addition, inter-rater agreement (nonweighted Cohen’s kappa) was determined for the binary classification of beta-amyloid positivity. A paired *t* test was used to evaluate any differences in subjective confidence between the STD and GM-EDGE binary classifications for each physician. The chi-squared test was used to test concordance between the STD and GM-EDGE binary decision frequencies for all five physicians. Values of *p* <0.050 were considered as statistically significant. Statistical analyses were performed using MedCalc, version 18.5, except for the calculation of Fleiss’ kappa [17].

## Results

The cohort of 121 consecutive patients consisted of 64 women and 57 men with a median age of 71 years (range 40–90 years). One patient showed significant movement in the late-phase, and the scan in this patient was excluded from analysis of the STD and GM-EDGE methods. In another patient, the early-phase images were too noisy to reliably delineate the brain boundary, and the scan in this patient was therefore excluded from analysis of the GM-EDGE method. In total, the scans from 120 patients were available for analysis of the STD method and from 119 patients for analysis of the GM-EDGE method.

Ten one-to-one physician pairs were generated. Cohen’s kappa values for inter-rater agreement for the five-point beta-amyloid positivity scale for each physician pair are shown in Table 1 for the STD and GM-EDGE methods. The median kappa value for the STD method was 0.70 (range 0.63–0.73), and for the GM-EDGE method was 0.80 (range 0.76–0.89). The ICC was higher for the GM-EDGE method (0.91, 95% CI 0.89–0.94) than for the STD method (0.84, 95% CI 0.79–0.88; non-overlapping 95% CIs for ICC indicate that the inter-rater agreement for the GM-EDGE method was significantly higher). Cohen’s kappa values for inter-rater agreement for the binary beta-amyloid classification for each physician pair are shown in Table 2 for both methods. The median kappa value for the STD method was 0.82 (range 0.73–0.93), and for the GM-EDGE method was 0.93 (range 0.90–0.97). Fleiss’ fixed-marginal multirater kappa value was higher for the GM-EDGE method (0.93, 95% CI 0.87–0.99) than for the STD method (0.82, 95% CI 0.77–0.88).

**Table 1:** Linearly weighted kappa values for the five-point measure of beta-amyloid positivity using the STD and GM-EDGE methods

Physician pair	Kappa value	
	STD method	GM-EDGE method
A – B	0.733	0.887
A – C	0.696	0.863
A – D	0.698	0.834
A – E	0.698	0.802
B – C	0.722	0.841
B – D	0.663	0.793
B – E	0.710	0.770
C – D	0.631	0.781
C – E	0.674	0.757
D – E	0.680	0.755

**Table 2:** Nonweighted kappa values for the binary classification of beta-amyloid positivity using the STD and GM-EDGE methods

Physician pair	Kappa value	
	STD method	GM-EDGE method
A – B	0.833	0.933
A – C	0.767	0.933
A – D	0.833	0.966
A – E	0.800	0.933
B – C	0.800	0.932
B – D	0.933	0.933
B – E	0.865	0.899
C – D	0.800	0.933
C – E	0.733	0.899
D – E	0.865	0.933

**Fig. 1:**
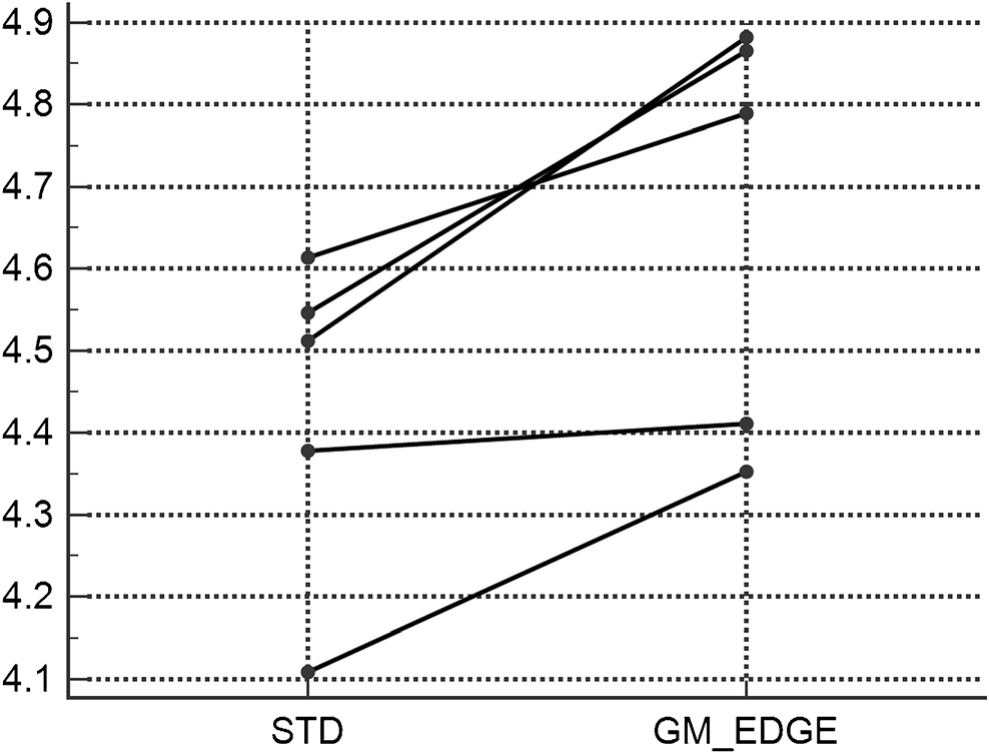
Arithmetic mean scores on the five-point scale for subjective confidence in the binary classification of amyloid positivity. Subjective confidence in the reading of four physicians was significantly higher with the GM-EDGE method than with the STD method

The arithmetic mean scores on the five-point scale for subjective confidence in the binary classification for individual physicians were 4.51, 4.55, 4.61, 4.11 and 4.38 for the STD method, and 4.88, 4.87, 4.79, 4.35 and 4.41 for the GM-EDGE method (Fig. 1). The subjective confidence in the reading of four physicians was significantly higher with the GM-EDGE method than with the STD method (*p* < 0.001, *p* < 0.001, *p* = 0.006, *p* < 0.001). The subjective confidence in the reading of one physician without previous experience with amyloid FMM-PET was nonsignificantly higher with the GM-EDGE method (*p* = 0.717).

**Fig. 2:**
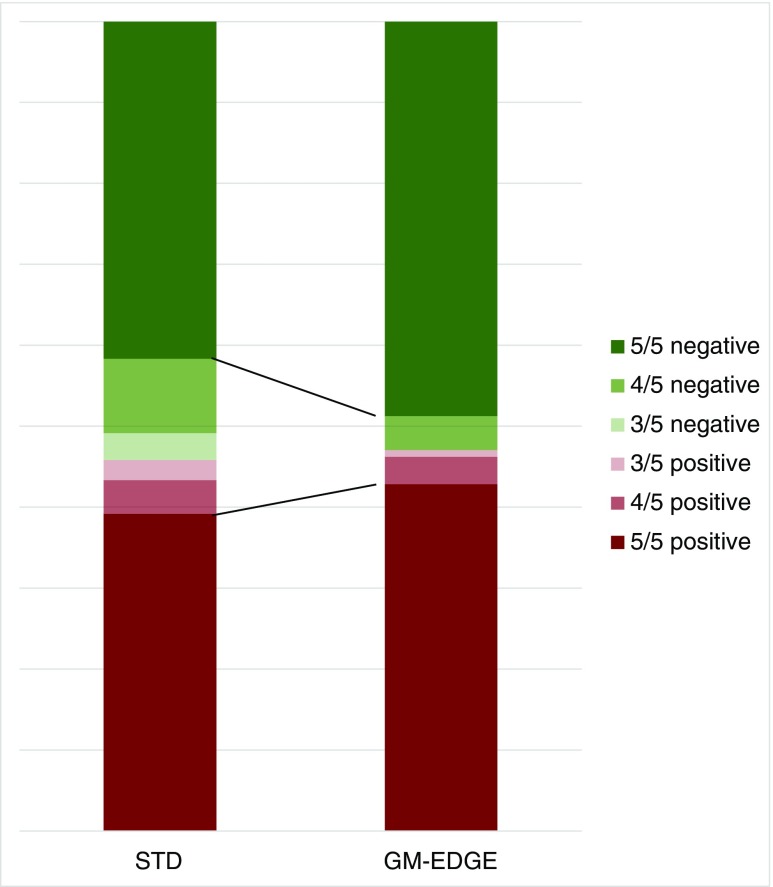
Frequencies of concordant binary results. Agreement is clearly better with the GM-EDGE method than with the STD method

Six subgroups of patients were created according to concordance in the binary classification among the five physicians. Observed counts for the STD and GM-EDGE methods are presented in Table 3 and Fig. 2. Using the STD method, all five physicians expressed the same decision in 97 of 120 patients (80.8%; 47 positive and 50 negative). Using the GM-EDGE method, all five physicians expressed the same decision in 109 of 119 patients (91.6%; 51 positive and 58 negative). The difference in frequencies was statistically significant (*p* = 0.016).

**Table 3:** Frequencies of concordant binary results

Concordance	Observed counts	
	STD method	GM-EDGE method
5/5 positive	47	51
4/5 positive	5	4
3/5 positive	3	1
3/5 negative	4	0
4/5 negative	11	5
5/5 negative	50	58

## Discussion

**Fig. 3:**
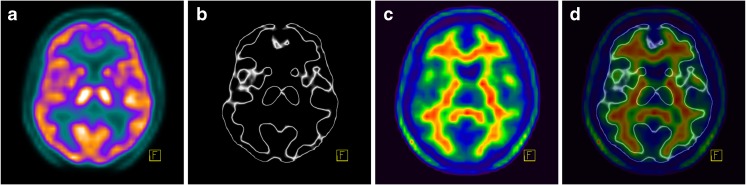
FMM PET imaging in a 53-year-old man with a low pretest probability of dementia. **a**, **b** Early-phase images presented in the “Warm Metal” (**a**) and “Edges” (**b**) colour scales. **c** The late-phase image shows a normal distribution of FMM. **d** Overlay of images **b** and **c** clearly shows no pathological uptake in the grey matter. This finding is not consistent with the presence of beta-amyloid

**Fig. 4:**
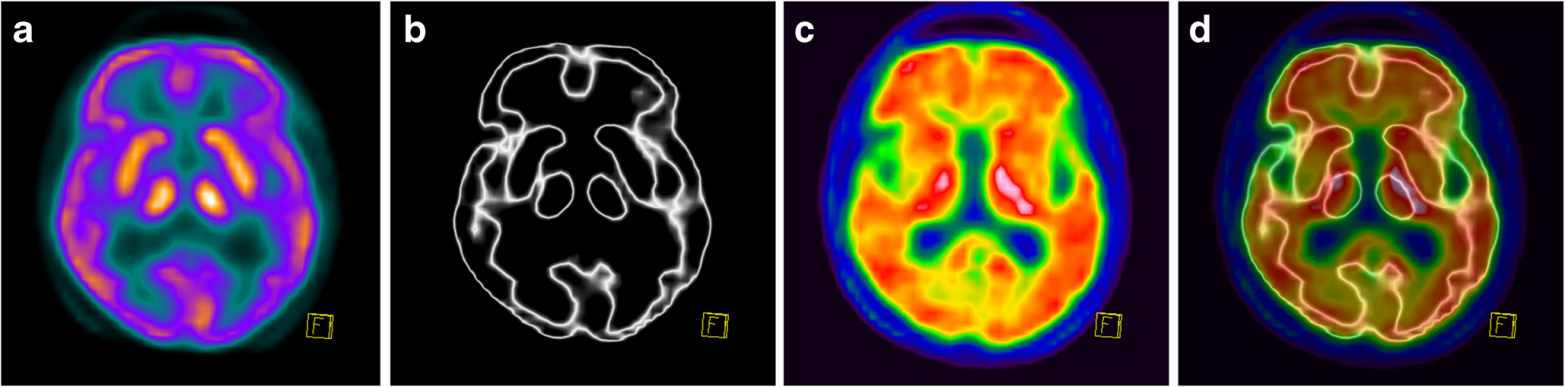
FMM PET imaging in a 62-year-old woman with a high pretest probability of Alzheimer disease. **a**, **b** Early-phase images presented in the “Warm Metal” (**a**) and “Edges” (**b**) colour scales. **c** The late-phase image shows a clearly abnormal distribution of FMM. **d** Overlay of images **b** and **c** clearly shows increased uptake in the grey matter. This finding is consistent with the presence of beta-amyloid

**Fig. 5:**
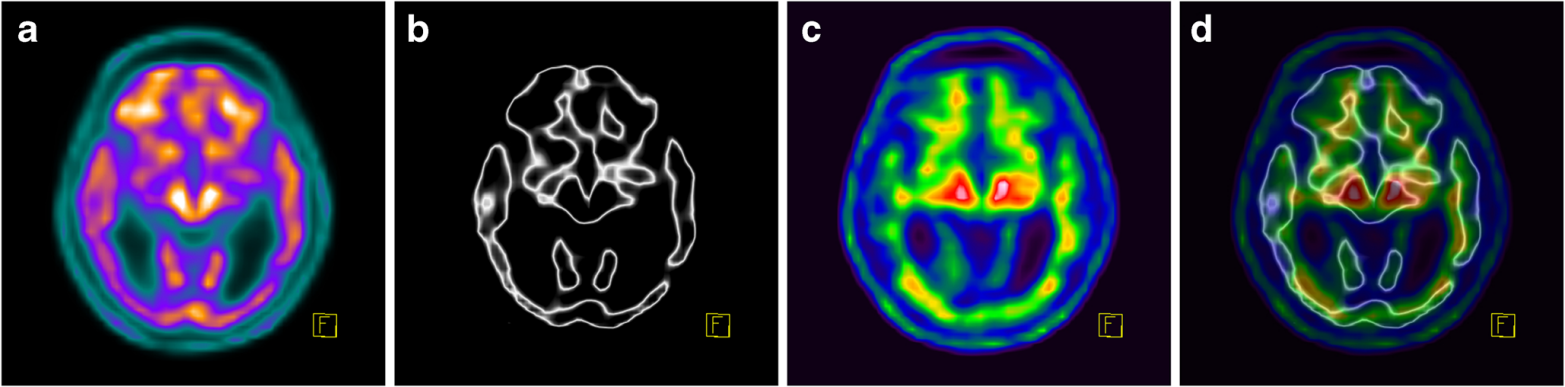
FMM PET imaging in a 72-year-old man with hydrocephalus, brain atrophy and dementia. **a**, **b** Early-phase images presented in the “Warm Metal” (**a**) and “Edges” (**b**) colour scales. **c** The late-phase image evaluated with respect to the thin grey matter ribbon is indeterminate, especially in the temporoparietooccipital areas. **d** Overlay of images **b** and **c** shows demarcation of the grey matter and is assessed as no pathological uptake in the grey matter. This finding is not consistent with the presence of beta-amyloid

Visualization of the border between white and grey matter using the GM-EDGE method enabled the easy distinction between normal nonspecific FMM uptake in the white matter and pathological uptake in the grey matter, consistent with the presence of beta-amyloid. Figures 3, 4 and 5 show FMM PET imaging in three example patients demonstrating the benefits of the use of the GM-EDGE method.

Brain biopsy/necropsy is considered the gold standard for determining the presence of beta-amyloid in the brain, but it is not commonly performed in the clinical setting and thus was not available in our cohort of patients. We were therefore not able to define the diagnostic accuracy of the methods. We did, however, evaluate inter-rater reliability for the GM-EDGE and STD methods in determining the presence of beta-amyloid. The median Cohen’s kappa value among the five physicians was 0.82 for the binary classification using the STD method. This result is consistent with kappa values in the range 0.73–0.82 reported by Harn et al. [18], who evaluated the agreement among three experienced raters in the reading of 55 florbetapir PET scans, and with the kappa values reported by Buckley et al. [19], who found that most values were greater than 0.80. Yamane et al. [20] found a slightly higher kappa value of 0.89 with ^11^C-based Pittsburgh compound B, which may be explained by the fact that 57 normal controls were included in the test cohort of 162 patients. Patients without cognitive impairment might exhibit lower variability leading to higher kappa values than patients with various levels of cognitive impairment, as those in our cohort.

Inter-rater reliability was significantly higher with the GM-EDGE method than with the STD method. The median Cohen’s kappa values for the binary classification among the five physicians were 0.82 with the STD method and 0.93 with the GM-EDGE method. This excellent inter-rater agreement is comparable with those reported by Harn et al. [18], who improved image reading with the addition of quantitative information extracted with the software MIMneuro (kappa 0.88–0.96). Together with the improvement in kappa values using the GM-EDGE method, there was also a significant decrease in the frequency of discordant reports among the physicians. Therefore, the use of the GM-EDGE method may improve consensus reading without any special software.

We evaluated the reports of five nuclear physicians, two experienced (>90 reports), two with no experience except the required training, and one with minimal experience with ten FMM PET/CT reports before starting the retrospective evaluation. For the binary classification of beta-amyloid positivity, inter-rater reliability was comparable between experienced physicians A and B and between physicians D and E without any experience with the STD method (Cohen’s kappa 0.83 and 0.87, respectively) and with the GM-EDGE method (Cohen’s kappa 0.93 and 0.93, respectively). This is a promising finding with respect to the reliability of beginners in reading of FMM PET/CT.

We also tested the subjective confidence of raters in the binary classification of beta-amyloid positivity. This result should be viewed with caution because possible bias might have been introduced by the subjective expectations of the authors, and by the possible scepticism of some physicians concerning the introduction of a new approach. Nevertheless, the physicians showed a higher degree of confidence in the GM-EDGE method.

At the time that FMM PET was introduced into clinical practice in our department, we did late-phase PET registration with common MRI sequences as recommended in the SPC [15]. The white/grey matter borders were not well demarcated on fused images, and photon spillover from white to grey matter complicated assessment. On the other hand, both early-phase and late-phase PET datasets are based on the same physical principle and therefore exhibit the same spatial resolution and the same intrinsic artefacts including the same photon spillover from grey to white and white to grey matter. Thresholding both datasets can easily compensate for spillover and facilitate assessment. This is a clear advantage over image fusion with MRI.

We assume that the GM-EDGE method would show similar performance in dual-phase PET with other beta-amyloid-seeking tracers. We have also confirmed the feasibility of this method in a group of more than 200 florbetapir PET scans (unpublished data).

Adequate evaluation of beta-amyloid PET data using both the STD and GM-EDGE methods requires the cooperation of patients in keeping their head still during data acquisition. Slight head movement during the late phase blurs white matter activity into the grey matter and may lead to false-positive findings. Similarly, slight head movement during the early phase results in fuzzy, enlarged grey matter. For correct interpretation, a check of movement on the cine display in all three planes and selection of a motion-free period for the final data reconstruction is essential.

### Conclusion

We introduce a simple method for beta-amyloid PET evaluation that is easily applicable to routine clinical practice without specialized software. This method exhibits excellent inter-rater agreement (median Cohen’s kappa 0.93) that is significantly better than that for the STD method of evaluation. The increased diagnostic sensitivity, specificity and overall accuracy of the new method needs to be confirmed in multicentre trials based on histologically confirmed two-phase beta-amyloid PET datasets.
